# Cell-specific expression of Epac2 in the subventricular and subgranular zones

**DOI:** 10.1186/s13041-019-0537-1

**Published:** 2019-12-23

**Authors:** Hyunhyo Seo, Kyungmin Lee

**Affiliations:** Department of Anatomy, School of Medicine, Kyungpook National University, 680 Gukchaebosang-ro, Jung-gu, Daegu, 41944 Republic of Korea

**Keywords:** Epac2, Ventricular-subventricular zone, Subgranular zone, Adult neurogenesis

## Abstract

**Aim:**

cAMP signal transduction cascade activation is important in regulating neurogenesis in adult rodents by increasing the proliferation of newborn cells. Although the ventricular-subventricular zone (V-SVZ) and subgranular zone (SGZ) both contain large populations of neural stem/precursor cells; it remains unclear whether an alternative target of cAMP, the exchange protein directly activated by cAMP (Epac2), is involved in adult neurogenesis in the V-SVZ and SGZ. Here, we investigated the cell-specific expression of Epac2 protein in the V-SVZ and SGZ of the adult mouse brain.

**Methods:**

Immunohistochemical analyses were performed using antibodies against Epac2, glial fibrillary acidic protein (GFAP), doublecortin (DCX), and beta-catenin, to examine the co-localization of Epac2 protein and neural stem/precursor cells in the V-SVZ and SGZ in three 8-week-old male mice.

**Results:**

In the V-SVZ of the lateral ventricle, most GFAP-positive adult neural stem cells (NSC, defined as type B cells) and 75% of DCX-positive migrating neuroblasts (type A cells) expressed Epac2 proteins. Ninety-three percent of beta-catenin-positive ependymal cells (type E cells), which are in direct contact with NSCs and the ventricles, also expressed Epac2 protein. Similarly, in the SGZ of the hippocampus, Epac2-immunopositive signals were shown by 83% of GFAP-positive radial-glia-like NSCs (type 1 cells), 86% of DCX-positive transiently amplifying cells (type 2 and type 3 cells), and 71% of DCX-positive immature neurons. The present data suggest that a PKA-independent cAMP signaling pathway via Epac2 may be party to adult neurogenesis in the V-SVZ and the SGZ.

## Main text

**Fig. 1:**
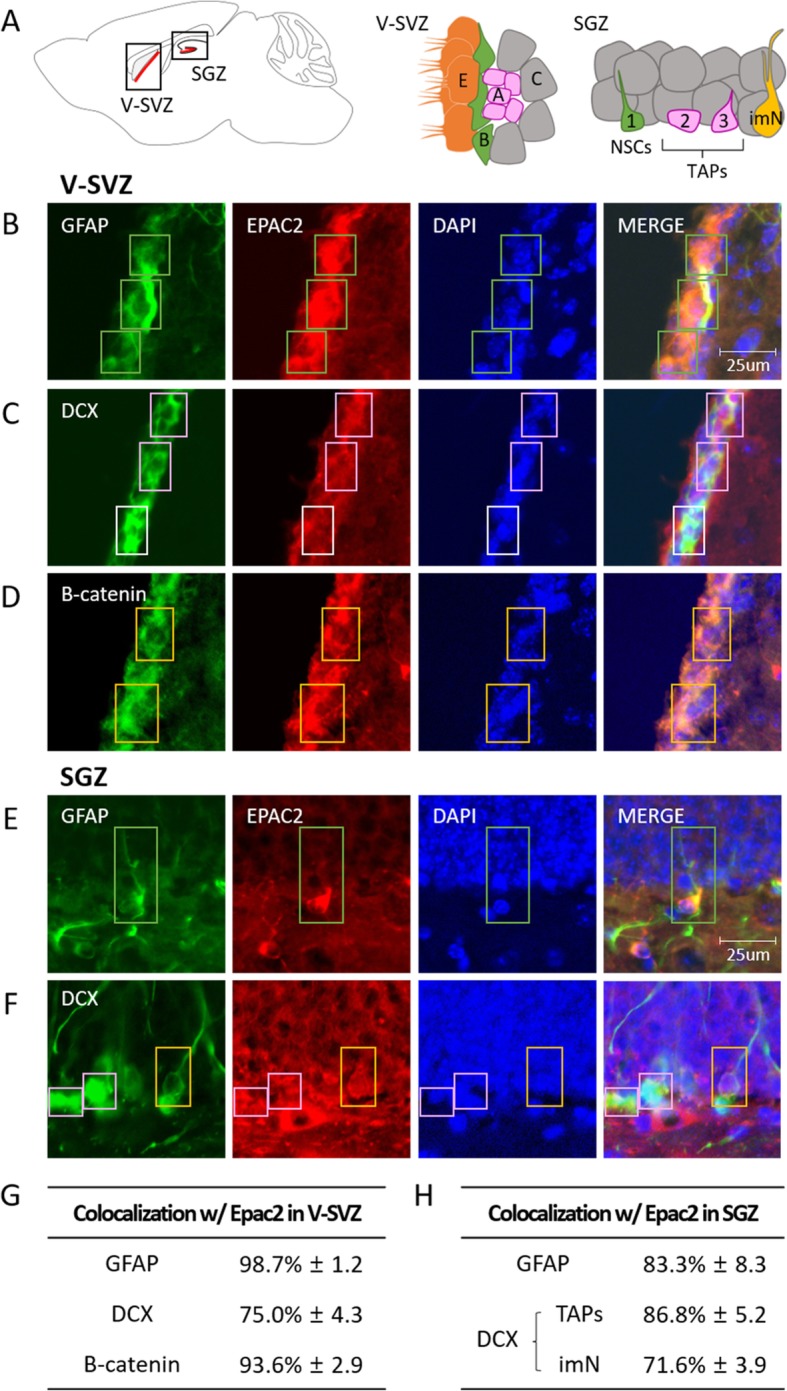
Colocalization of Epac2-immunopositive signals with NSCs, migrating neuroblasts, or ependymal cells in the V-SVZ of adult mice; and with NSCs, TAPs, or immature neurons in the SGZ. **a**. Overview of the V-SVZ and SGZ. Left panel, sagittal section through a mouse brain. Middle panel, cellular composition of the V-SVZ. A, type A cell; B, type B cell; C, type C cell; E, type E cell. Right panel, cellular composition of the SGZ. 1, type 1 cell (NSCs); 2, type 2 cell; 3, type 3 cell; imN, immature neurons. **b**-**d**. Representative immunofluorescence images of the V-SVZ. Epac2-immunoreactive signals are observed in the GFAP-positive NSCs (**b**, green boxes), DCX-positive neuroblasts (**c**, pink boxes), and beta-catenin-positive ependymal cells (**d**, yellow boxes). The white box shown in “**c**” indicates a neuroblast showing non-colocalization. **e**-**f**. Representative immunofluorescence images of the SGZ. GFAP-positive radial glia-like stem cells (**e**, green box), DCX-positive TAPs (**f**, pink boxes), and a DCX-positive immature neuron with one strong branching (**f**, yellow box) express Epac2 protein. Scale bars, 25 μm. **g**. Colocalization rates of Epac2-immunopositive cells with each of the cell marker protein-positive cells in the V-SVZ. H. Colocalization rates of Epac2-immunopositive cells with each type of cell marker protein-positive cell in the SGZ. **g** and **h**, Values in tables are presented as mean ± SEM.; *n* = cell numbers (See Materials and Methods)

In the adult mammalian brain, neural stem cells (NSCs) are retained in two regions, the ventricular-subventricular zone (V-SVZ) of the forebrain and the subgranular zone (SGZ) of the hippocampus (Fig. 1a, left) [1, 2]. The V-SVZ in the walls of the lateral ventricles contains a subpopulation of cells with astroglial properties (type B cells) that express glial fibrillary acidic protein (GFAP). These cells function as NSCs, giving rise to intermediate progenitor cells (type C cells), which in rodents, predominantly produce the migrating neuroblasts (type A cells) that mature into new neurons destined for the olfactory bulb (Fig. 1a, middle) [3]. In the SGZ of the dentate gyrus, NSCs also correspond to astroglial cells, which have a radial process that traverses the granule cell layer. These cells, known as type 1 progenitors [4] or radial glia-like cells [5], generate transiently amplifying progenitor cells (TAPs) including type 2 cells and migratory neuroblasts (type 3 cells), which amplify but subsequently exit the cell cycle ahead of maturation into granule neurons (Fig. 1a, right) [6]. Among the different intracellular signal transduction cascades, the PKA-dependent cAMP-cAMP response element-binding protein pathway is involved in hippocampal neurogenesis [7]. Interestingly, a recent study suggested that a PKA-independent alternative cAMP signal transduction pathway via an exchange protein directly activated by cAMP 2 (Epac2) also affects hippocampal adult neurogenesis by regulating progenitor cell proliferation [8]. Another study using Epac2 knockout mice [9] showed that Epac2 contributes to astrocytic differentiation in neural precursor cells [10]. However, there is little direct evidence of cell-specific expression of Epac2 in the V-SVZ and SGZ.

To address this issue, we conducted immunohistochemistry using antibodies against Epac2, GFAP to label NSCs, doublecortin (DCX) to label neuroblasts in the V-SVZ or TAPs and immature neurons in the SGZ, and beta-catenin to label ependymal cells. Double immunofluorescence staining of coronal brain sections of three 8-week-old male mice with Epac2 and one of GFAP, DCX, or beta-catenin showed that Epac2 protein was expressed in germinal zones of both the V-SVZ and SGZ. In the V-SVZ, Epac2-immunopositive signals were observed in over 98% of GFAP-positive NSCs (Fig. 1b, green box and 1Gg) and 75% of DCX-positive migrating neuroblasts (type A cells; Fig. 1c, pink box and g). The Epac2 signals were also co-localized with 93% of the beta-catenin-positive ependymal cells (type E cells; Fig. 1d, yellow box and g) that were in immediate contact with GFAP-positive NSCs and cerebrospinal fluid (CSF), which contains soluble factors that could modulate the behavior of NSCs [11]. When viewed en face from the ventricular side, the small apical endings of NSCs are surrounded by a rosette of ependymal cells with large apical surfaces [12], and ependymal cells are known to help maintain the molecular composition of the apical compartment of NSCs by propelling the CSF with their multiple motile cilia [13]. Although we could not evaluate the expression of Epac2 in the type C cells that are represented by less than 10% of the cells in the V-SVZ [14], these findings suggest that the NSCs, neuroblasts, and ependymal cells related to neurogenesis express Epac2 protein in the V-SVZ. Similar to the V-SVZ, Epac2-immunoreactive signals were also colocalized with 83% of GFAP-positive radial glia-like stem cells (type 1 cells; Fig. 1e, green box and 1H), although the total number of GFAP-positive NSCs was much less in the SGZ than in the V-SVZ (see the Additional file 1; Materials and Methods). DCX-immunopositive signals were found in TAPs, including type 2 cells (Fig. 1f, left pink box) and migrating neuroblasts with short processes (type 3 cells; Fig. 1f, right pink box), as well as in postmitotic immature neurons with strong dendritic branching (imN; Fig. 1f, yellow box) in the SGZ at the hippocampus. In the SGZ, we observed Epac2-immunoreactive signals in 86% of DCX-positive TAPs and 71% of immature neurons (Fig. 1h). These findings are consistent with the previous data published by Zhou et al. suggesting that Epac2 gene deletion induced the most obvious decrease in the number of DCX-positive cells with short processes and DCX-positive cells with one strong dendrite branching [10]. Taken together, our findings indicate that Epac2 protein may play an important role in both proliferative and postmitotic stages during neurogenesis in the SGZ.

In conclusion, the Epac2 protein that mediates PKA-independent cAMP signaling may be involved in neurogenesis in the V-SVZ and SGZ of adult mice, and further study to address the specific role of Epac2 in adult neurogenesis is needed.

## Supplementary information

**Additional file 1.** Materials and Methods.

## Data Availability

All materials are available in the Additional file 1 (Materials and Methods).

## Abbreviations

CSF: cerebrospinal fluid
DCX: doublecortin
Epac2: exchange protein directly activated by cAMP 2
GFAP: glial fibrillary acidic protein
NSCs: neural stem cells
SGZ: subgranular zone
TAPs: transiently amplifying progenitor cells
V-SVZ: ventricular-subventricular zone
