# Supplementary material for: Cell-specific expression of Epac2 in the subventricular and subgranular zones
Source: Mol Brain. 2019 Dec 23;12:113. doi: 10.1186/s13041-019-0537-1 (PMC6929441; doi:10.1186/s13041-019-0537-1)
Supplement: Supplementary file 1 — Additional file 1. Materials and Methods. [file 13041_2019_537_MOESM1_ESM.docx]

**Materials and Methods**

**Animals**

All experiments and animal protocols were performed in accordance with the guidelines for the Care and Use of Laboratory Animals of Kyungpook National University. All animals were maintained under standard conditions with a temperature of 21°C ± 1°C, humidity of 53% ± 3%, and a 12 hour light/dark cycle (light from 10 a.m./dark from 10 p.m.). Three 8-week-old male mice (C57BL/6J) were used for the experiments.

**Tissue preparation**

Mice were first anesthetized with Avertin solution (125 mg/kg) composed of 2,2,2-tribromoethanol (T48402, Sigma-Aldrich, MO, USA) and the same volume of 2-methyl-2-butanol (152463, Sigma-Aldrich). They were then transcardially perfused with ice-chilled 4% paraformaldehyde (PFA) in PBS (pH 7.4) and the brain was removed and immediately post-fixed in 4% PFA in PBS for 12 hours. After subsequent overnight dehydration in 30% sucrose in PBS, the brain tissue was embedded with optimal cutting temperature compound and sectioned into 30-μm coronal sections using a cryo-microtome (CM 3050S, Leica). The sections were then mounted onto glass slides (silane coating Muto-glass, 5116-20F, Muto pure chemicals CO., LTD., Tokyo, Japan).

**Immunohistochemistry**

Brain tissues were rinsed three times in 1× PBS and then treated with 0.1% Triton X-100 (T8787, Sigma-Aldrich) in PBS containing 4% normal goat serum (s-1000, Vector laboratory) for one hour at room temperature for blocking and permeabilization. The following primary antibodies were used for immunostaining: mouse anti-GFAP antibody (1:500, G6171, Sigma-Aldrich), goat anti-DCX antibody (1:100, sc8066, Santa Cruz, TX, USA), mouse anti-beta-catenin antibody (1:100, #610154, BD Transduction Laboratory, NJ, USA), and Abcam rabbit anti-Epac2 antibody (1:200, ab124189, Abcam, Cambridge, UK) for double staining with anti-DCX, and custom-made rabbit anti-Epac2 antibody (1:50, AbFrontier, Seoul, South Korea) for double staining with anti-GFAP or anti-beta-catenin. After primary antibody incubation at 4°C for 16 hours, tissue samples were washed three times in PBS containing 0.1% tween-20 (p9416, Sigma-Aldrich). Samples were then treated with secondary fluorescent-probe labeled antibodies (Alexa, Molecular probes, OR, USA) as follows: anti-mouse Alexa 488 (A11001) for anti-GFAP and anti-beta-catenin, anti-goat Alexa 488 (A11055) for anti-DCX, and anti-rabbit Alexa 488 (A10040) for both anti-Epac2 antibodies. Images were collected on a Zeiss (Thornwood, NY) confocal microscope using a 40× objective lens with a 10× eyepiece, and images with a further 2× magnification were acquired by zooming in on each immune-positive cell for analysis. The numbers of brain slices and total numbers of each type of cell-marker-positive cells used for the quantification of colocalization of cells with Epac2 signals were: 4 slices and 133 cells for GFAP, 6 and 127 for DCX, and 4 and 66 for beta-catenin were used in the V-SVZ, while in the SVZ, 5 slices and 15 cells were used for GFAP, and 5 and 207 for DCX.
